# Knowledge, attitude, intention, and religion aspect toward fertility preservation among obstetrics and gynecology residents in Indonesia: A cross-sectional study

**DOI:** 10.18502/ijrm.v18i1.6199

**Published:** 2020-01-27

**Authors:** Achmad Kemal Harzif, Mila Maidarti, Melisa Silvia, Ana Mariana, Heidi Dewi Mutia, Budi Wiweko

**Affiliations:** ^1^Department of Obstetrics and Gynecology, Faculty of Medicine Universitas Indonesia, Dr. Cipto Mangunkusumo Hospital Jakarta, Indonesia.; ^2^Indonesian Reproductive Medicine Research and Training Center (INA- REPROMED) Faculty of Medicine Universitas Indonesia, Dr. Cipto Mangunkusumo Hospital Jakarta, Indonesia.

**Keywords:** Knowledge, Attitude, Intention, Fertility preservation.

## Abstract

**Background:**

The lack of knowledge from healthcare providers regarding fertility preservation will certainly affect the patient's knowledge, attitude, behavior, and also perspective. Obstetrics and Gynecology (OB/GYN) residents may most likely be the first line professionals to integrate fertility preservation technologies into their practice which plays an important task in giving an understanding of the relationship between age and fertility for patients.

**Objective:**

This study aims to assess OB/GYN resident knowledge and beliefs regarding age-related fertility decline, intentions, and religion aspect toward fertility preservation.

**Materials and Methods:**

A cross-sectional study was conducted on 92 Indonesian OB/GYN residents at the Cipto Mangunkusomo Hospital between November and December 2017. Data were collected using a questionnaire which had four sections, knowledge, attitude, intention, and religion aspect toward fertility preservation.

**Results:**

The majority of participants believed that an OB/GYN should encourage discussions about potential childbearing desires (96.74%) and age-related fertility decline (94.57%) with patients, of which 79.34% believed that these discussions should be part of a woman's annual health examination. Cancer patients are likely to undergo oocyte cryopreservation than people who choose career as priority. From the religion aspect, fertility preservation options such as sperm, oocyte, embryo, and ovarian cortex cryopreservation were accepted by most residents with varied religions, while oocyte and sperm donor methods were unacceptable (48% and 57%, respectively) because of the belief that oocyte/sperm should only be given to legitimate partners, but many still do not know that oocyte and sperm donor were prohibited by all religions.

**Conclusion:**

Age-related fertility decline and frozen egg storage should be discussed during annual woman wellness examinations by OB/GYN specialists.

## 1. Introduction

According to estimates from the World Health Organization (WHO) International Agency for Research on Cancer (IARC), the world age-standardized incidence rate shows that there are 165 new cancer cases for every 100,000 females in the world (1). Oncology Society of Indonesia in 2010 reported that 63.67% of cancer cases were that of women of which 31.4% were under 45 yr old (2). More people with cancer are expected to face long-term side effects of the treatment, especially with regard to their reproductive system (3). Studies by Momen and co-workers suggest that fertility becomes one of the markers of quality of life to be of concern to cancer patients who still want to have children (4). The American Society of Clinical Oncology recommended that physicians promptly build communication with patients especially with cancer about fertility preservation methods and potential complications of reproduction function regarding the age-related decline (5).

Studies by Adams E and colleagues in England suggest that oncologists assume that preservation of the reproductive function is important. However, 87% of them need more information about the preservation of reproductive function beyond sperm storage. Only about 38% of them discussed preservation options with their patients in writing (6).

ESHRE reviewed that OB/GYN residents and general practice play an important task in giving an understanding of the relationship between age and fertility for patients which integrating with new evidence-based medicine and technologies (7). The lack of knowledge from healthcare providers regarding the preservation of reproductive function certainly affects the patient's knowledge, attitude, behavior, and perspective. A study in Brazil in 2017 showed that the knowledge and attitudes of the patients on the preservation option of the reproductive function are still very poor (8). In Indonesia, no studies have observed the effect of knowledge, attitudes, behavior, and religion perspectives on OB/GYN residents regarding preservation of reproductive function.

Therefore, this study aims to determine the profile of knowledge, attitude, intentions, and religion aspects on the preservation of reproductive function on the OB/GYN residents in Indonesia.

## 2. Materials and Methods

### Measures 

This cross-sectional study was conducted on 92 Indonesian OB/GYN residents in the Cipto Mangunkusumo Hospital between November and December 2017. Data were collected using questionnaires. All items of the questionnaire were developed to measure knowledge, attitude, intentions, and religion aspects regarding age-related fertility decline and oocyte cryopreservation.

### Instrument design 

The questionnaire was based on Yu and colleagues' and had been tested before. The instruments measured fertility awareness and the clinical experiences of the authors in the fields of OB/GYN, reproductive endocrinology, psychology, and anthropology (9). The survey included demographic background questions, questions about residents' knowledge toward awareness of fertility issues, questions about residents' attitudes toward fertility issues and the methods of storing frozen oocytes, and questions about residents' intentions on concerning the need for discussion or supporting oocyte cryopreservation for different patient situations. Participants were also asked about their religion perspective on the preservation of reproductive functions, they were asked whether the preservation of reproductive functions such as oocytes freezing, sperm freezing, embryos freezing, ovarian cortex freezing, oocyte donors, and sperm donors were allowed or not allowed based on their religion perspective.

### Procedures

In total, 100 OB/GYN residents were asked to fill out all items of the questionnaire. Five residents refused to fill the questionnaire. After the questionnaires were collected back, three of them were excluded because they did not complete all items of the questionnaire. Finally, 92 OB/GYN residents were included in this study as participants.

### Ethical consideration

This study has been approved by the ethics committee of the Faculty of Medicine, University of Indonesia (reference number: 985/UN2.F1/ETIK/2017).

### Statistical analysis 

The primary analysis was descriptive. Frequencies and proportions were summarized for characteristics and each questionnaire items. Bivariate test was used for religion perspective on reproductive preservation. The statistical analyses were performed using the Statistical Package for the Social Sciences, version 20.0 (SPSS Inc., Chicago, IL, USA). In this study, we used three analyses test to compare the data, such as (1) Binomial test with p < 0.05 for Residents' attitudes toward fertility issues and the use of oocytes cryopreservation, (2) Goodness of fit Chi-square test with p < 0.05 for Likelihood of concerning the need for discussing or supporting oocyte cryopreservation for different patient situations, and (3) Contingency coefficient test with p < 0.05 for Religion perspective on reproductive preservation.

## 3. Results

### Sample of characteristics

Ninety-two OB/GYN residents participated in this study demographic characteristics revealed that more than half of the residents were less than 30 yr old and significantly higher percentage of respondents were women (60.87%). More than half of the OB/GYN residents were Muslim (61.95%), and most of the respondents (26.08%) intended to pursue general practice as an OB/GYN specialist in the future and have no intention to be a trainee (Table I).

### Knowledge of OB/GYN residents on the awareness of fertility issues

**Figure 1:**
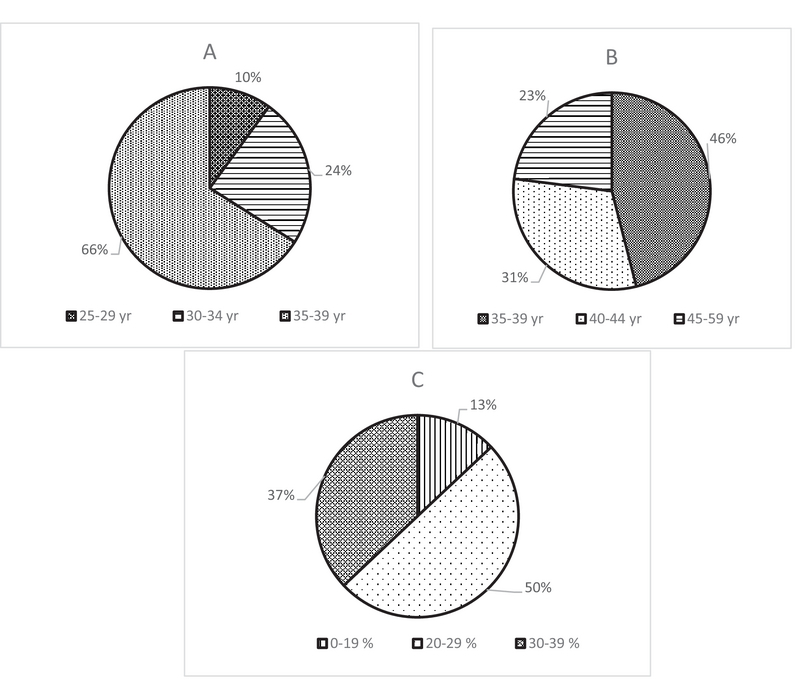
(A) Residents' knowledge about the age of women's ability to get pregnant becomes declines slightly. (B) Residents' knowledge about the age of women's ability to get pregnant becomes meaningful decreased. (C) Residents' knowledge about the average chance of IVF therapy to be successful just after a one-time treatment.

As shown in Figure 1A, more than half of the OB/GYN residents (66%) overestimated the age when fertility declines slightly to be 35–39 yr, and as shown in Figure 1B, almost half of the residents (46%) overestimated the age when fertility declines markedly to be 35–39 yr. Also residents overestimated the overall chance of success in having a child after undergoing one IVF treatment cycle, as half of the OB/GYN residents (50%) believed that the success rate was 20–29% (Figure 1C).

### Attitudes of OB/GYN residents toward fertility issues and methods of storing frozen oocytes

Our results showed that almost all participants OB/GYNs should initiate counseling with their patients about childbearing desires (96.74%) and age-related fertility decline (94.57%). About 79.34% of participants thought that an annual woman wellness examination with an OB/GYN specialist should present a discussion about age-related fertility decline since this encourages women in deciding their future potential reproduction. Moreover, participants thought that OB/GYNs should establish a discussion about the option of frozen oocyte storage with their female patients (86.95%). Nearly half of the respondents thought this option should be discussed in the part of an annual woman wellness examination (Table II).

### Intentions of OB/GYN residents toward the need for discussing or supporting oocyte cryopreservation for different patient situations

As shown in Table III, there is a difference in the recommendation between patient situation. OB/GYN residents were very likely to discuss oocyte cryopreservation with patients who had received a cancer diagnosis, regardless of whether that patient was 25 or 35 yr of age. Almost half of the residents highly recommended and were very likely to discuss oocyte cryopreservation in a 25-yr-old patient with a cancer diagnosis (38.04%). In contrast, residents were either somewhat or very unlikely to discuss oocyte cryopreservation with patients who wanted to pursue a career before starting a family in a 25-yr-old patient (16.30%).

### Religion aspect of OB/GYN residents on the preservation of reproductive functions

Table IV shows the difference between the Muslim, Christian, and Catholic residents on the preservation of reproductive function. Half of the residents in different religion thought that freezing oocyte, freezing sperm, and freezing embryo were allowed. There was no significant difference between the three religions. However, there was a significant difference in the oocyte and sperm donors (p = 0.008 and p = 0.001). Half of the Islamic residents thought that oocyte (54.39%) and sperm donations (61.40%) were not allowed. In contrast, most of the Christian and Catholic residents did not know that oocyte and sperm donations were allowed or not.

**Table 1:** Characteristics of the sample


**Characteristics**		**Total (n = 92)**
Age		
	≤ 30	57 (61.96)
	> 30	35 (38.04)
Religion		
	Islam	57 (61.96)
	Christian	16 (17.39)
	Catholic	14 (15.21)
	Buddha	2 (2.17)
	Hindu	1 (1.08)
	Unknown	2 (2.17)
Gender		
	Woman	56 (60.87)
	Man	34 (36.95)
Trainee plan		
	Fetomaternal	18 (19.56)
	Fertility and endocrine-immunology reproduction	15 (16.30)
	Urogynecology	2 (2.18)
	Oncology gynecology	10 (10.86)
	Obstetrics social	3 (3.26)
	Other majors	0 (0)
	General practice	24 (26.08)
	Others	12 (13.05)
	Unknown	8 (8.71)
Data presented as n (%)		

**Table 2:** Residents' attitudes toward fertility issues and the use of oocytes cryopreservation


**Questions**	**Yes**	**No**	**P-value**
Should an OB/GYN specialist discuss the patient's desire to have a child?	89 (96.74)	3 (3.26)	0.000*
Should an OB/GYN specialist discuss with the patient about age-related fertility decline?	87 (94.57)	5 (5.43)	0.000*
Should the discussion about the natural age-related fertility decline become part of a woman's annual examination with an OB/GYN specialist?	73 (79.35)	19 (20.65)	0.000*
Should an OB/GYN open a discussion about the choice of frozen oocyte storage with the patient?	80 (86.95)	12 (13.04)	0.000*
Should the discussion about frozen oocyte storage become part of a woman's annual examination with OB/GYN specialist?	62 (67.39)	30 (32.60)	0.001*
Data presented as n (%)			
*Significant (p < 0.05), using binomial test			

**Table 3:** Likelihood of concerning the need for discussing or supporting oocyte cryopreservation for different patient situations


**Characteristics**	**Highly do not recommend**	**Give little recommendation**	**Recommend**	**Highly recommend**	**P-value**
25-yr-old patient with cancer diagnosis	4 (4.34)	12 (13.04)	41 (44.56)	35 (38.04)	0.00*
25-yr-old patient with career priority	15 (16.30)	33 (35.86)	38 (41.30)	6 (6.52)	0.00*
35-yr-old patient with cancer diagnosis	7 (7.60)	20 (21.73)	42 (45.65)	23 (25)	0.00*
35-yr-old patient with career priority	9 (9.78)	33 (35.86)	37 (40.21)	13 (14.13)	0.00*
Data presented as n (%)					
*Significant p < 0.05, using Goodness of fit Chi-square test					

**Table 4:** Religion perspective on reproductive preservation


**Preservation of reproductive function**	**Islam**			**Christian**			**Catholic**			**P-value**
	**Allowed**	**Not allowed**	**Don't know**	**Allowed**	**Not allowed**	**Don't know**	**Allowed**	**Not allowed**	**Don't know**	
Freezing oocyte	39 (68.42)	2 (3.51)	16 (28.07)	10 (62.50)	2 (12.50)	4 (25.00)	10 (71.43)	0 (0.00)	4 (28.57)	0.54
Freezing sperm	38 (66.67)	2 (3.51)	17 (29.82)	10 (62.50)	2 (12.50)	4 (25.00)	10 (71.43)	0 (0.00)	4 (28.57)	0.53
Freezing embryos	33 (57.89)	2 (3.51)	22 (38.60)	8 (50.00)	2 (12.50)	6 (37.50)	6 (42.86)	1 (7.14)	7 (50.00)	0.6
Ovarian cortex freezing	41 (71.93)	1 (1.75)	15 (26.32)	9 (56.25)	1 (6.25)	6 (37.50)	8 (57.14)	0 (00.00)	6 (42.86)	0.49
Oocyte donors	9 (15.79)	31 (54.39)	17 (29.82)	4 (25.00)	1 (6.25)	11 (68.75)	2 (14.29)	4 (28.57)	8 (57.14)	0.008*
Sperm donors	10 (17.50)	35 (61.40)	12 (21.10)	4 (25.00)	1 (6.25)	11 (68.75)	2 (14.29)	5 (35.71)	7 (50.00)	0.001*
Data presented as n (%)										
*Significant (p < 0.05), using contingency coefficient test										
Buddhist, Hindu, and others were not included due to small population										

## 4. Discussion

In our best knowledge, this is the first study to examine the attitudes, knowledge, intentions, and religion aspect toward fertility preservation among Indonesian OB/GYN residents, especially in Jakarta. Most of residents who completed the survey believed that OB/GYNs should establish discussion about childbearing intentions. Otherwise, 95% of participants said that OB/GYN should discuss about age-related fertility decline with female patients, where 79% said that these discussions should be a part of an annual woman wellness examination. A large study that collected data from 79 countries reported that people who postponed childbearing also have lack knowledge of age-related fertility decline; however, about 56.9% of participants who reach higher fertility knowledge were women, educated and living in Very High Human Development Index (VHHDI) countries (10). Many women have already known that physicians and healthcare providers have much information on fertility and reproductive health, but they reported that they never discuss the influence of age on their ability to conceive in detail (6). In spite of showing a positive attitude for giving enough information for patients about fertility decline by physicians, most of OB/GYN residents had misled about fertility decline. Dunson *et al* reported that advancing age of women was strong correlated with decreased of fertility. This previous study highlighted that patients and physicians should aware about fertility decline (11). In this study, we found that almost half of the residents were not well-informed about these basic reproductive facts while OB/GYNs place in the first line to distribute adequate reproductive knowledge, particularly fertility information for society. A previous study found a significant positive correlation that suggested between intention to delay childbearing in a group who got fertility information. Therefore, better education and curriculum about age-related decline in female fertility should be offered in OB/GYN residency programs in Indonesia (12).

In addition in this study resident were misinformed about the success rate of ART based on age-related infertility. Half of the residents (50%) think that the success rate of IVF is high. Practitioners must be educated that the IVF successful rate decrease in women who postponing to conceive from age 20 to 35 (13). Many women have a misperception that the success rate of ART does not relate to age. Only 11.7% of women aged 41-42 had a live birth with ART and the chance decrease in women aged 43-44 years old, which is only 4.5% success rate. But in women aged under 35 have 41.5% could have a chance had a live birth (14). The women aged 40s have a less chance to have live birth with ART than younger women. Therefore, a high educated practitioner should inform the patient and correct this misperceptions.

When OB/GYN residents in this study were asked whether they should initiate discussions regarding oocyte cryopreservation with patients, more than three quarters (87%) believed that OB/GYNs should open a discussion with patient, and more than half (67%) reported that it should be a part of an annual woman wellness examination. A study in 2013 revealed that the oocyte of the 183 women who undergone oocyte cryopreservation cycle with mean age was 38 have reduce the oocytes quality and potential reproductive, Only one-third women discussed with gynecologist before the oocyte cryopreservation procedure, 79% of the women in the study wish they had undertaken the procedure before (15). The current study revealed women aged 31 have greater potential than older women who undergoing oocyte cryopreservation, the OB/GYN resident tends to start a discussion about oocyte cryopreservation at women aged 31. A decision analysis model was conducted by Mesen (2015) showed that women who undergo oocyte cryopreservation before 34 years old have highest probability to achieve live birth outcome (16). OB/GYN has to consider any possibilities factor while inform patients about the decline of fertility and oocyte cryopreservation, the pregnancy plan might be influenced by other factors beyond patient control (15). The physician has to be sensitive and respect to patients' reproductive autonomy. Ideally, to achieve adequate counseling, OB/GYN should initiate counseling about the highest probability of reproductive potential that could be maximized at 20-30 years old. Knowing the attitudes and knowledge of the physician about fertility preservation is important. In Germany, 120 oncologists believe that fertility preservation was an important issue, 40% of the physicians discussing the issues with patients routinely, and only half who understanding of it (17). A study in Japan found that breast cancer specialist was more positive attitudes toward fertility preservation, they have improved the interdisciplinary communication between physicians and infertility specialists by discussing fertility preservation with patients (18). In this study, residents are more likely to support oocyte preservation for cancer women than age-matched patients. Therefore, further study is needed to assess the financial covering of fertility preservation technologies that might have an impact on attitudes toward and using of oocyte cryopreservation.

Religion perspectives in this study show that most Islamic residents thought that freezing oocyte, sperm, embryos, and ovarian cortex were allowed. In contrast, ovum and sperm donor were not allowed. It could be explained by the principle of assisted reproductive technology in Islamic perspective. The treatment of infertility in Islam is allowed only if it involves the preservation of procreation. The sperm, ovum, and uterus have to come from the same couple that are legally married and during their contract of marriage. Marriage could be ended by death or divorce. If marriage ends, female partners are not allowed to use sperm cell from their ex-husbands, and likewise male partners are not allowed to use ovum from their ex-wives. When sperm or ovum are from anonymous donors, like ovum or sperm donors, the procedure is absolutely forbidden by Islamic law (19, 20). As in Islam, Judaism allowed all the techniques of ART only if the sperm and oocyte are from the wife and husband. Religion has an influence on women authorities in the reproduction aspect, such as fertility treatment. Protestant, Anglican, and other Denominations accept ART treatment, but the Vatican does not accept that procedure. In Catholic, the procreation as not a process of manufacture. The Catholic view of procreation is inseparable from married sexual intercourse, because of the respect of the “gift” and sanctify of created life is fundamental (21). The problem of views about “the good life” is that they rest on a religious or naturalistic presupposition that not all participants necessary to share in the secular view (22). The American Society for Reproductive Medicine (ASRM) declared that freezing of embryo should no longer be considered experimental in 2012 (23). The secular views were rejected by critical appraisals that the reproduction aspect is a personal thing to which one has a right (22). Additionally, IVF with donor oocyte or donor sperms is not allowed by the law in Indonesia (24).

The knowledge about the different religious perceptions related to the reproduction health problem, especially fertility preservation is important for a practitioner who practices reproduction techniques. Dilemmas associated with fertility therapies and ethical considerations should be addressed because this field continues to evolve. Therapy of fertility should consider the age and marital status, religion or ethical objection to freezing of embryo, treatment, and type of cancer, the risk to benefit ratio of delaying treatment, and prognosis after treatment (25). Greater awareness about psychosexual and psychosocial morbidity associated with cancer patients' attitudes, emotions, cancer-related infertility, and the choices of having children is needed (26). The loss of fertility might affect sexuality, identify and role expectations, and the pursuit of intimacy and marriage not only inability to have children (27).

Finally, women have to make their choice about fertility preservation. It must be highlighted that the decision is not made by only the women, but include the male partners (28). Some studies revealed that men also overestimate the age-related to fertility declines (29). Furthermore, a desire for having a child are related to whether a male partner desires or not (30). Therefore, discussion of fertility decline and oocyte cryopreservation must include men not only women to give the full range of reproductive options and adequate information of each option (31).

## 5. Conclusion 

Our study revealed that there is a highlight critical need to improved OB/GYN resident education about issues related to age-related fertility decline and the use of oocyte cryopreservation for both medical and elective reasons at the Cipto Mangunkusumo Hospital. Most residents assume that age-related fertility decline and oocyte cryopreservation should be discussed during annual examinations. From the religious perspective, it is shown that preservations of reproductive function were mostly accepted except oocyte donor and sperm donor.

##  Conflict of Interest 

The authors declare that they have no conflict of interest.
